# Hybrid Polymer Composites of Bio-Based Bast Fibers with Glass, Carbon and Basalt Fibers for Automotive Applications—A Review

**DOI:** 10.3390/molecules25214933

**Published:** 2020-10-25

**Authors:** Anjum Saleem, Luisa Medina, Mikael Skrifvars, Lena Berglin

**Affiliations:** 1 Department of Applied Logistics and Polymer Sciences, University of Applied Sciences Kaiserslautern, 67659 Kaiserslautern, Germany; Luisa.Medina@hs-kl.de; 2 Swedish Centre of Resource Recovery, Faculty of Textiles, Engineering and Business, University of Borås, 50190 Borås, Sweden; Mikael.Skrifvars@hb.se; 3 Department of Textile Technology, Faculty of Textiles, Engineering and Business, University of Borås, 50190 Borås, Sweden; Lena.Berglin@hb.se

**Keywords:** bast fibers, basalt fibers, mechanical analysis, synthetic fibers, carding, compression molding

## Abstract

Composites with reinforcements based on bast fibers such as flax, hemp and kenaf offer many advantages such as weight reduction, improved specific impact, flexural, acoustic properties, and balanced performance to cost that can be achieved by properly designing the material composition. Their position is well established, especially in the nonstructural automotive applications. However, in structural applications of composites, their mechanical property profile is not comparable to the dominant reinforcements such as glass and carbon fibers. The low mechanical properties of these composites could be improved by hybridization that involves adding high-performance fibers to the bast fiber composites that could improve the low mechanical performance of the bast fiber composites. The review presented in this article provides an overview of the developments in the field of hybrid polymer composites composed of bio-based bast fibers with glass, carbon, and basalt fibers. The focus areas are the composite manufacturing methods, the influence of hybridization on the mechanical properties, and the applications of hybrid composites.

## 1. Introduction

The use of natural fiber reinforced polymer composites especially in the automotive industry is established and well documented [1,2,3,4]. This is because of the unique properties of natural fibers—such as low density, high specific strength and stiffness, non-abrasive to the equipment during processing, abundant availability, renewability, sustainability, and environmental friendliness [5,6]. Their specific modulus is comparable and at times even better than the glass fibers [6]. Furthermore, they manifest effective damage tolerance in the composites. Despite their technical, ecological, and economic benefits, they have certain drawbacks such as non-uniform and non-reproducible properties due to soil and weather conditions, and the way they are extracted. Characteristics such as higher flammability, limited mechanical properties, incompatibility with hydrophobic polymer matrices, thermal degradation at high processing temperatures, and poor resistance to moisture are also challenges [3,7,8]. For highly demanding structural applications, their mechanical performance cannot compete with those of the synthetic fibers such as glass or carbon fibers in the composites industry [4]. Because of the increasing eco-awareness along with the strict regulations of the legislative authorities, there is a significantly enhanced interest in natural fiber reinforced polymer composites not just in semi-structural but also in structural applications [9,10]. However, several technical considerations have to be addressed before their widespread acceptance in scientific and commercial fields.

Glass fiber reinforced plastics have proven to meet the demands in structural applications in automobiles because of their good strength, relatively good interfacial fiber–matrix adhesion, and well-developed manufacturing methods [4]. Carbon fibers are mostly used when the requirements of strength and durability are higher [11,12]. They are commonly used in many automotive applications such as wheels and parts of sports cars and engine covers; the BMW electric cars use a carbon fiber body. The major shortcomings of synthetic fibers are their high costs (especially for carbon fibers), dependency on fossil fuel and accumulation in the environment due to non-degradability. Their processing is quite challenging because of the involvement of toxic chemicals and gases [13,14]. Table 1 presents briefly the mechanical, ecological and health aspects of the synthetic, basalt, and bast fibers.

Over the past few years, there is an increasing interest in using several reinforcing components in a single composite matrix (ceramic, steel, or polymer). This approach is called hybridization and provides an opportunity to a more versatile use of composites with improved properties in advanced applications. It is a lucrative way to combine various fibers as reinforcements so that the composites exhibit better properties compared to a composite with only one individual reinforcing constituents. Benefits which can be achieved are reduced cost, higher modulus and strength, corrosion resistance, and even improved thermal stability. It overcomes the limitations of one reinforcement type by combining with another type having better performance [19]. The mechanical properties and other characteristics of bast fiber-reinforced composites can clearly be improved by hybridization with high strength fibers.

The aim of this paper is to review the literature contributions about the hybridization of bast fibers with high-performance, synthetic (glass and carbon), and natural (basalt) fibers in polymer matrices and the influence of their hybridization on the composite properties. The following sections present a short introduction of the hybrid composites, the factors affecting hybridization, a literature review, and an overview of the applications of hybrid composites.

### 1.1. Hybrid Composites

Hybrid composites have two or more different fiber types simultaneously as reinforcement in one matrix [20] and the choice of the hybrid reinforcements depends upon which shortcoming of the composite needs adjustment in a particular application. The properties of the hybrid composites depend upon several factors such as fiber content, fiber length and orientation, fiber–matrix interaction, fiber stacking sequences, and the properties of individual constituents of the composites. A main factor is the proportions of the different fiber types in the reinforcement. The properties of hybrid composites with two different reinforcements can be predicted by a rule of mixtures [21,22,23].

$$P_{h}=P_{1\;}V_{1}+\;P_{2\;}V_{2}$$

where:

*P_h_* = property to be investigated
*P*_1_ = property of first component
*V*_1_ = volume fraction of first component
*P*_2_ = property of second component
*V*_2_ = volume fraction of second component

When the experimental results of a certain property are better than the results from the rule of mixture, it is called positive hybridization and vice versa.

Hybridizing natural fibers with high strength fibers in polymer composites provides an excellent opportunity to improve their property profile for structural applications. Several contributions have reported the hybrid composites of natural cellulosic fibers with synthetic fibers [15,24]. The major advantages that cellulosic/glass or carbon hybrid composites offer are high strength to weight ratios and less amount of synthetic fibers that reduce their environmental impact.

**Figure 1:**
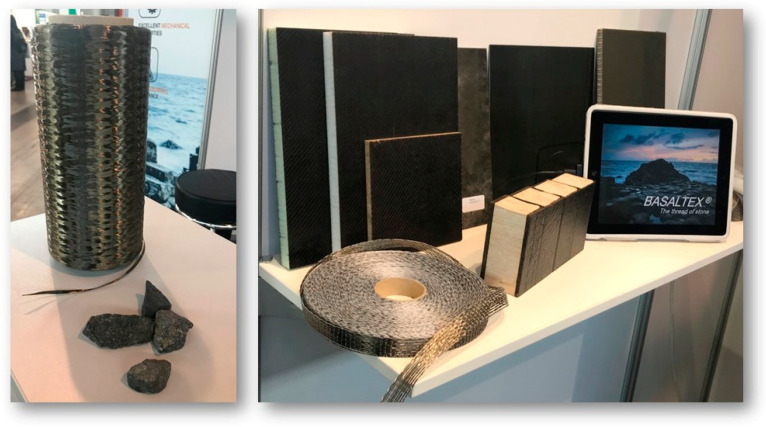
Various basalt products (fibers, rocks, laminates) from Basaltex displayed at Composites Europe, Stuttgart, Germany 2019 (photo by authors at the event).

Another attractive reinforcement that is slowly making its mark in the composites is basalt. Basalt fibers (Figure 1) are natural inorganic fibers made from basaltic rocks.

Initially, their applications were restricted to space and military operations. Since 1991, they have been introduced in commercial applications [25,26] and are often considered as an alternative to the glass fibers. Table 2 shows a comparison of the chemical composition of glass and basalt fibers. Compared to the glass fibers, they have almost 5% higher density, 15–20% higher tensile and compressive strength and about 23% higher modulus of elasticity (Table 1). Furthermore, they have a temperature resistance above 500 °C, high chemical resistance, anti-corrosion, anti-frictional properties, less abrasive than glass fibers [27], as well as low moisture and water absorption [25,28].

Additional advantages over glass fibers are their better environmental profile and no health hazards during processing. They are inert and have been classified as non-toxic and non-carcinogenic. In Europe and the US, they are available as safe material. However, care in handling is recommended [17,30]. Compared to carbon and glass fibers, basalt fibers came relatively late on the market. Therefore, there is a dearth of literature about their composites. In the Publons publications database, there are for basalt fibers reinforced composites only 180,086 records compared to the records for glass (230,430) and carbon (458,377) fiber-reinforced composites [31]. However, the use of basalt fibers in composites is rapidly increasing because of their excellent technical properties, renewability and sustainability characteristics [30,32,33]. The hybridization of bio-based bast fibers with basalt fibers provides natural fine-tuning of the mechanical properties of the composites since basalt fibers are natural high strength fibers.

### 1.2. Factors Affecting Hybridization of Natural Fibers in Polymers

The properties of natural fiber reinforced polymer composites are influenced by certain factors associated with the natural fibers. These factors are briefly discussed here.

#### 1.2.1. Hydrophilicity

Natural fiber reinforced composites tend to take up moisture from the environment because of the hydrophilic nature of the natural fibers. They can absorb up to 5–15 wt % of moisture. Moisture absorption can cause dimensional variations, poor fiber–matrix interaction and poor fiber dispersion in the matrix [34]. These issues with natural fibers result in composites with inferior mechanical properties [35]. There are solutions to control the hydrophilicity by fiber treatments with alkali, acetylation, bleaching, grafting, or coating. Other techniques to improve fiber–matrix interfacial adhesion are the use of compatibilizers such as silanes, maleated polypropylene (MAPP), and titanates.

#### 1.2.2. Poor Thermal Resistance

Natural cellulosic fibers are mostly used in low-temperature applications because of their low thermal stability. Their maximum processing temperature is usually 200 °C. This limits their applications because they cannot be processed by all the manufacturing methods, especially those involving high-temperature exposure [36]. While designing the natural fiber composites, the investigation of its thermal stability is a crucial requirement that defines the processing methods and applications of NF products.

#### 1.2.3. Naturally Existing Variations and Biodegradability

The mechanical, physical, and chemical properties of cellulosic fibers depend strongly on the harvest, climate, location, weather conditions, and soil characteristics. These are naturally occurring irregularities and almost impossible to control [37]. The major attraction of these fibers is their biodegradability. They degrade very easily by biological, chemical, or mechanical methods. However, in certain applications, it is required that the product has a longer service life and therefore their biodegradability must be controlled. In such cases, the biodegradability could be controlled by chemically blocking the hydroxyl group in the cellulose molecule on the cellulosic fibers [38].

### 1.3. Literature Review

**Figure 2:**
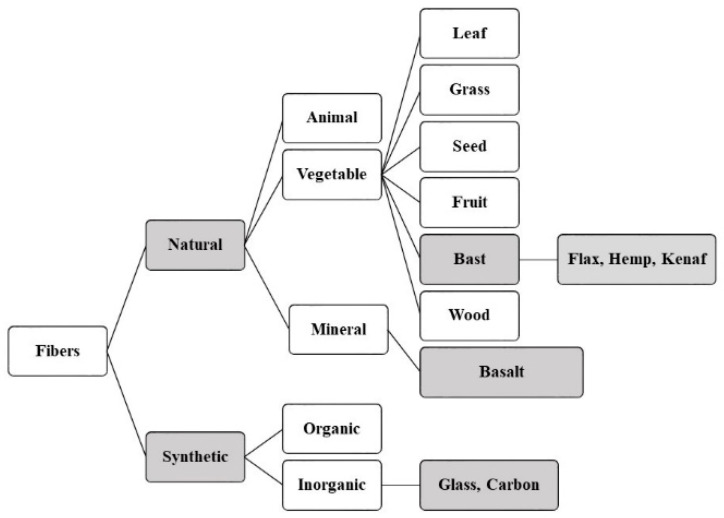
Classification of fibers ([4,6] modified illustration).

The classification of fibers in Figure 2 shows their availability in a wide variety. Not all natural fibers have the optimal mechanical performance to be used as reinforcements. Besides wood particles, bast fibers such as flax, hemp, and kenaf and leaf fibers such as sisal are the most used fibers in the composites.

**Figure 3:**
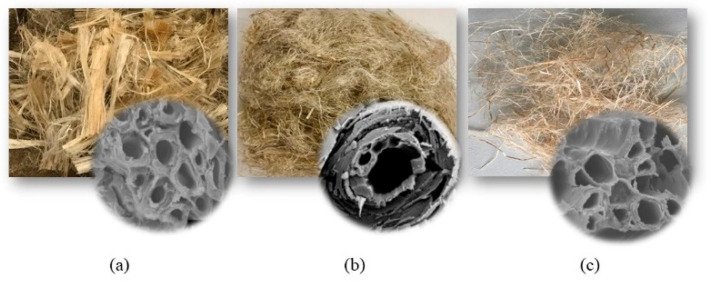
Short (**a**) kenaf, (**b**) flax and (**c**) hemp fibers and cross section of a fiber bundle (modified illustration [6]).

Bast plants are known for their long, strong bundles that form the outer portion of the stalk and bast fibers have often high strength in the bast portions. Their use is common in non-woven fabrics, molding compounds and composite components especially in the automotive industry as door panels, headliners, seat backs, etc. They have very similar microscopic structure as can be seen in Figure 3. The choice of a single fiber for a certain application depends upon factors such as availability or part geometry, flax for example is flexible and preferred for complicated geometries.

A great deal of work reported about the bast fiber hybrid composites deals with the study of their mechanical properties, influence of fiber length, fiber loading, fiber–matrix interaction, fiber arrangement, and fiber or matrix modification. When it comes to polymers, the common matrices of choice are epoxy, unsaturated polyester, phenolic, vinyl ester, and polyurethane resins. Whereas the thermoplastic hybrid composites are based of polypropylene, polyethylene, polyvinyl chloride, polycarbonate, and natural rubbers. The following section presents a brief overview of the hybrid composites of bast fibers with carbon, glass, and basalt fibers.

#### 1.3.1. Hybrid Bast/Carbon Composites

The properties of bast fibers are considerably different compared to the carbon fibers. In the literature, there are not many contributions likely because of the significant differences between stiffness and price of the bast and carbon fibers [39]. Despite this, hybrid bast/carbon fiber composites could offer the benefits of high strength and modulus and good impact behavior that might be of interest in applications where impact performance coupled with low weight is of particular interest [15]. This section provides an overview of the research contributions about bast/carbon hybrid composites.

##### Flax/Carbon

Flax fibers are one of the strongest natural fibers and are used frequently in the automotive sector. Their hybridization with carbon fibers could generate composites with excellent damping and improved strength. Fiore et al. [7] studied the influence of unidirectional carbon fabric layer on the mechanical performance of bidirectional flax/epoxy composite. The authors aimed to improve the mechanical properties of the flax fiber reinforced laminates (FFRP) making them suitable for structural applications, especially in the automotive industry. They replaced a bidirectional flax fabric with unidirectional carbon fabric. The composites were tested for their flexural and tensile properties. The analysis showed that the hybrid flax/carbon composites had a significant increase in properties compared to only FFRP composite. The flexural modulus and strength of FFRP-C composite were about 221% and 110% higher than FFRP. The hybrid (FFRP-C) composites showed promising properties to be used in structural applications.

The sandwich structure construction of carbon and flax fibers was further investigated by Bagheri et al. [40] as bone implants. They determined in their study the mechanical properties of a new hybrid carbon/flax composite. The flax layers were sandwiched between the carbon layers. The composites showed better flexural properties compared to the tensile properties, which is an advantage for applications where adequate bending stiffness is needed. The composite manifested promising properties to be used in orthopedic fracture fixation.

Dhakal et al. [41] prepared and compared fiber laminates with two types of flax/carbon hybrid laminates either cross-ply (CP) or unidirectional (UD) flax fabric laminates in epoxy resin. The composites were tested for their water absorption, tensile properties, thermogravimetric, and flexural properties. The performance of UD hybrids was better than the cross-ply laminates. They concluded that the flax fibers contribute to the improvement of toughness properties by crack propagation and carbon fibers contribute to the improvement of the thermal stability, water absorption, strength, and stiffness of the composites.

Several contributions have presented studies on the mechanical properties, hydrothermal characteristics and fiber–matrix interactions of the natural fiber reinforced composites. However, in structural applications, it is very important to get an insight into the damping properties of these materials, which are required to reduce vibration and to avoid fatigue fracture. Assarar et al. [42] investigated the effects of stacking sequences and hybridization on the damping properties of flax–carbon epoxy composites. Several composites were prepared with various stacking sequences of carbon and flax. The dynamic properties of the materials were investigated using beam test specimens and an impulse technique. Finite element analysis was used to model the damping to evaluate the dissipated energies in the layers of composites. It was concluded that the position of flax layers is important in defining the bending stiffness and damping properties.

Guen et al. [43] quantified the damping coefficient of flax and hybrid flax/carbon epoxy composites prepared by compression molding and vacuum bagging. The results explained the impact of hybridization on the damping coefficient, tensile, and flexural properties of the composites. The elastic modulus-damping model was validated successfully with the results. They applied the rule of hybrid mixtures for hybrid composites and observed a positive hybridization effect except for flexural properties.

Using composites in structural applications especially in automotive requires tests that are representative of real-life experience such as the study of a material’s behavior in an accident situation. The falling weight impact method is normally performed on the materials to obtain information about energy absorption, the morphology of damage, and its propagation to test the suitability of materials for structural applications. Nisini et al. [44] presented a quite complex ternary hybrid of flax, carbon, and basalt in an epoxy matrix. The focus of the study was to analyze the falling weight impact performance of the composites. The carbon layers were always kept at the external side. It was suggested that keeping the flax layers in the center of the laminate generates a locking effect that effectively contributes towards damage dissipation and the sequence of stacking plays a key role to achieve the benefits of hybridization.

Sarasini et al. [39] produced hybrid composites of flax and carbon in epoxy with different stacking sequences. Flexural and falling weight impact testing was done to characterize the composites. The sequence flax—carbon—flax showed superior properties than only flax composites. They observed that the presence of flax in the outer layer acts as a hindrance and prevents crack propagation.

##### Hemp/Carbon

Hemp is one of the best natural fiber candidates because of its excellent mechanical properties. It requires easy environmental conditions for growth and shows a very low consumption of fertilizers and pesticides compared to the other natural fibers. Therefore, hemp can be grown easily around the world [45,46]. The use of hybrid hemp composites is emerging in structural applications. Scutaru et al. [47] studied the mechanical properties of polyester-based hybrid hemp/carbon laminates manufactured by hand layup method. The plies sequence was carbon—hemp—carbon—hemp. The composites were characterized for their impact properties at various impact speeds. The failure analysis at 4 m/sec impact speed showed the crack propagation from the place where the intender hits to the back of the test panel. The crack was only significant at the front side where the intender hits. The intensity of the cracking decreased significantly and the back of the sample showed only a slight projection and minor rips This concludes an excellent stiffness of the hybrid composite. Such materials are attractive for the automotive industry where good impact properties are required.

Ramesh et al. [48] studied hybrid hemp/carbon composites with polyester resin for tensile, flexural, impact, shear, and water absorption properties. The elastic behavior and the prediction of the composites strength was done by finite element analysis. The theoretical and experimental findings were in agreement. The alkali-treated hemp fibers performed better than the untreated fibers.

Pinto et al. [49] focused on the design and production of hybrid laminates of carbon and hemp fibers with epoxy resin. The laminates were manufactured by hand layup and vacuum compression molding. Three-point bending, interlaminar shear, damping, and impact test at various energy levels from 5–20 J were carried out. The stacking sequence of reinforcements is very important to define and control the properties of the composites. It was concluded that the hybridization of hemp and carbon has the potential to allow the development of new load bearing structures because it allows the creation of laminates with tailorable properties in and out of plane direction according to the relative position of carbon and hemp layers.

##### Kenaf/Carbon

Kenaf cultivation yields are comparatively higher than for flax and hemp providing a cost-effective raw fiber. They are cleaner due to water retting and show lesser content of shives or dust. Like other plant fibers they have low density, nonabrasive nature, biodegradability, and good mechanical properties and therefore, they are very attractive in the automotive sector.

Anuar et al. [49] studied the hybridization of carbon and kenaf in thermoplastic natural rubber. They studied the effect of fiber loading, fraction and treatment on the mechanical and thermal properties of the composites. The flexural properties increased with up to 15 vol % of the fiber content and then declined. The higher fiber content resulted in an increment of impact strength for both treated and untreated fibers composites. The dynamic mechanical analysis showed that the hybrid composites with untreated fibers exhibited higher storage, loss, and damping moduli compared to the composites with treated fibers. They observed that the fiber treatment resulted in poor dynamic mechanical properties and concluded that it could be because of factors such as low aspect ratio, random distribution of the fibers and incompatibility between carbon and kenaf fibers that led to the phase separation of fiber and matrix and affected the mechanical performance of the composites.

Mechanical properties of the composites are governed by the reinforcement and fiber–matrix interactions. To investigate further Bakar et al. [50] studied the impact properties of kenaf/carbon hybrid epoxy composites. The composites were manufactured in a hand lay-up process. The overall fiber content in the composites was 20 wt %. The effect of reinforcement in various ratios of kenaf/carbon was focused at the impact properties of the composite. The composites were prepared by both treated and untreated fibers in order to improve the fiber matrix interaction. Carbon fibers were treated with 100 kGy of gamma radiation and kenaf fibers with 4% NaOH solutions for the improvement of fiber–matrix interaction. They concluded that the increment of carbon fiber content and the use of treated fibers contribute to the improvement of impact properties of hybrid kenaf/carbon composites. The impact strength of the composites with treated fibers was about 26% higher than the untreated fiber composites.

Sapiai et al. [51] investigated the tensile and compressive properties of kenaf fiber reinforced and hybrid kenaf/plain weave carbon epoxy composites in the longitudinal and transverse direction related to the kenaf fiber direction. The composites were prepared by hand layup and vacuum bagging. The hybrid composites tested in the longitudinal direction showed an increase of 136% compressive strength, 58% compressive modulus, 160% tensile strength, and 65% tensile modulus. The properties of hybrid kenaf/carbon composites were significantly better than only kenaf fiber composites. The properties were higher in the longitudinal direction of the fibers because in this case they depend upon the tensile strength of the fibers, whereas in transverse direction they depend upon the shear properties of the matrix.

Aisyah et al. [52] studied the effect of various fabric materials and fabric counts on the properties of laminated woven kenaf/carbon fiber reinforced epoxy composites prepared by vacuum infusion technique. The weave pattern and the fabric count control the properties of the composites. It was concluded that the fabric structure, strength, and content significantly affects the properties. Plain fabric showed better adhesion with the composite compared to the satin fabric. SEM analysis of failure mode showed that the failure is caused by fiber pull out and voids. An increment of fiber volume and reduction of voids increases the tensile properties. The composites showed promising properties for applications in structural applications.

##### Jute/Carbon

Jute is a promising bast fiber having low cost, wide commercial availability and good thermal and insulation properties. Lenda et al. [53] investigated the water absorption behavior of hybrid jute/carbon epoxy composites. The focus of the study was to investigate the influence of the immersion time and the fiber content on the water absorption behavior. The analysis showed that the moisture absorption increased with the increasing fiber content in hybrid composites. It was found that the impact strength reduced with the increased moisture content and the reduction was larger in the specimen with high fiber content. The maximum impact strength was reduced from 104 to 84 kJ/m^2^, 138 to 114 kJ/m^2^, and 167 to 142 kJ/m^2^ for hybrid specimens with fiber volume fractions of 0.68, 0.58, and 0.47, respectively. It was observed that the main cause of failure in specimens with (higher) moisture content was delamination accompanied by fiber breakage.

Hybrid composites of jute/carbon in epoxy resin offering high modulus and strength combined with low cost and ability to damp vibration were analyzed further by Ashworth et al. [54] for their tensile and damping properties. The composites were prepared by the resin transfer molding process. The tensile modulus was almost double for hybrid composites (15.1 GPa) compared to the jute fiber composite (8.2 GPa). The analysis showed that the hybridization of low cost, sustainable jute with carbon fibers offers an economic and environment friendly alternative to carbon fiber reinforced composites with outstanding damping properties.

Sezgin et al. [55] studied the effect of different stacking sequences of jute and carbon fabric plies on the dynamic mechanical properties of the composites. The composites were manufactured by vacuum bagging technique. Each laminate had two plies of carbon and two plies of jute in varying positions of the layers. It was found that the composite with carbon fabric in outer layer showed the highest storage and loss modulus.

Ramana et al. [56] studied the development of new hybrid composites of jute and carbon fibers in epoxy resin prepared by hand layup process. The mechanical properties of the hybrid composites were promising to replace the carbon–epoxy composites without major loss of tensile and flexural properties. Furthermore, it was found that the hybrid composites had better ductility and impact strength.

Table 3 highlights the research contributions about the hybrid bast/carbon composites, their manufacturing methods, and properties analyzed.

#### 1.3.2. Hybrid Bast/Glass Composites

The stiffness of some of the bast fiber reinforced composites is found to be superior to the glass fiber reinforced composites. However, in terms of strength and impact properties, they achieve the target level only occasionally. The mechanical performance of bast fiber reinforced composites could be improved by replacing a part of bast fibers with glass fibers, which have high strength and reproducible characteristics [58]. This section provides an overview of research contributions on bast/glass hybrid composites.

##### Flax/Glass

Benevolenski et al. [59] studied the transverse perforation impact behavior of flax mat reinforced PP composites with additional discontinuous cellulose and glass fiber mat. The reinforcement content was 50 wt.% for all the composites. The resistance to perforation impact improved significantly by hybridization with cellulose and glass fibers. The improved resistance to perforation was also shown by Charpy impact strength data. This enhanced resistance was because of improved strength, which affected the failure behavior, as well. Although increased Charpy impact strength was associated with the increase in resistance to perforation, no universal correlation between these parameters could be established.

Morye et al. [60] studied the mechanical properties of glass/flax hybrid composites in a soybean oil-based thermoset matrix. The composites were tested for their flexural and impact strength properties. The mechanical properties were dependent on the glass/flax ratio and the arrangement of the fibers in the composites. Both fibers in the hybridization acted synergistically and the flexural and impact properties of the hybrid composites improved significantly.

Zhang et al. [23] studied mechanical performance of unidirectional flax and glass fiber composites with phenolic resin prepared by compression molding. The aim was to investigate the effect of hybridization on mechanical performance. The tensile properties increased by increasing the glass fiber content and the stacking sequence. Hybrid composites manifested higher fracture toughness and interlaminar shear stress, as well.

Barvarz et al. [61] investigated the mechanical and aging properties of flax/glass hybrid composites of PP. The results showed that the mechanical properties improved by increasing the glass fiber content. There was a significant improvement in the tensility, impact resistance, and hardness of the hybrid composites. However, the strain at yield and the elongation at break were almost unaffected by adding glass fibers. Glass fibers also enhanced the water resistance of the flax reinforced composite. Thermal aging at 85 °C showed that irrespective of filler type and content the composites were thermally resistant. The analysis of the composites by UV ageing test showed that the glass fibers accelerate the degradation of the PP matrix, but flax fibers can protect the composites. Furthermore, a partial least squares model was developed to correlate the properties of aged and unaged samples.

Saidane et al. [62] presented a hybrid composite of glass and flax fibers in the epoxy matrix. They reported a positive hybridization effect manifested by the increased tensile modulus and strength for hybrid composites. The composites were immersed in water at 55 °C for the analysis of water resistance and the acoustic emission coupled with scanning electron microscope was used to identify the typical damage mechanism. A statistical model was used for the identification in which parameters and classes were optimized. They evaluated the damage mechanism by hit number and acoustic energy. The results showed that even if the number of hits associated with fiber failure was less, their contribution to the failure was significant in terms of cumulative acoustic emission energy.

Calabrese et al. [63] studied the mechanical stability and durability of hybrid flax/glass epoxy composites. The aim was to use these materials for structural applications in the marine environment. The hybrid composites were compared with the individual glass and flax fiber reinforced composites, as well. All samples were tested for aging in a salt–fog environment for 60 days. The hybrid composites showed improved flexural strength, modulus and an improved aging resistance indicating a positive effect of hybridization.

Kumar et al. [64] studied the hybrid composites of biaxial glass and woven flax fabrics in vinyl ester resin. The composites with various stacking sequences were manufactured via vacuum-assisted resin transfer molding. The mechanical properties such as tensile, flexural and Charpy impact energy were better for hybrid composites. The tensile strength of pure glass fibers composite was lower than the hybrid glass/flax composite (85.16 vs. 143.21 MPa). The flexural and impact properties were significantly affected by the arrangement of stackings. The flexural strength of the composite with glass ply at the bottom was highest (305.46 MPa), whereas the composites with glass play on both ends showed high impact strength (0.145 J/mm^2^).

Paturel et al. [65] manufactured flax, glass and hybrid flax/glass vinylester composites by resin infusion technique. The composites were studied for their moisture absorption and impact properties. The hybrid composites manifested improved properties compared to the composites without hybridization indicating that hybridization is an effective strategy for improving the structural performance of natural fiber reinforced composites.

##### Hemp/Glass

Pantapulakkal et al. [66] presented their work on hybrid composites of short hemp and glass fibers in PP matrix. They prepared the composites by melt blending and studied their thermal, mechanical, and water resistance properties. All properties showed a significant improvement in the hybrid composites.

Shahzad [67] studied the impact and fatigue properties of hybrid hemp and glass composites in a polyester matrix. He observed an increase in the impact strength of up to 11% fiber volume. However, the fatigue sensitivity of the composites did not show any considerable change in the hybrid composites.

##### Kenaf/Glass

Davoodi et al. [68] compared the mechanical properties of kenaf reinforced epoxy composites with hybrid kenaf/glass epoxy composites. These composites were developed for intended use in automotive applications such as car bumper beams. They reported a positive hybridization effect on the tensile and flexural properties. The properties were further compared with the properties of a typical bumper beam material (a glass mat reinforced thermoplastic). It was found that the properties were comparable except for the impact properties, which were lower for the hybrid kenaf/glass composites. Nevertheless, the materials showed promising properties and the potential to replace glass fiber composites in structural applications.

Maleque et al. [69] investigated the flexural and impact properties of hybrid kenaf/glass reinforced unsaturated polyester composites. The matrix loading was always 70% volume fraction with a varying volume fraction of kenaf and glass fibers. The kenaf fibers were treated for three hours with a 6% sodium hydroxide solution. The composites were manufactured by the sheet molding process. The analysis of the composites showed that the hybrid composites with treated kenaf fibers (15/15 *v*/*v*) showed the highest flexural strength, whereas the untreated fiber composite with the same composition showed the highest impact strength. They concluded that the hybrid composites with kenaf and glass in 15% volume fraction are the most promising materials for structural applications in automotive, aerospace, or sports.

Afdzaludin et al. [70] studied the synergistic effect of kenaf/glass hybridization in an unsaturated polyester composite. The composite was manufactured by the sheet molding compound process. The analysis showed that the flexural properties for the composition with 15/15 *v*/*v* hybrid fiber mat were most optimal.

Ismail et al. [71] analyzed the effect of kenaf/glass hybrid composites in epoxy resin. The composites were analyzed by low-velocity impact testing. The best properties were shown by hybrid composites with 75% glass fiber and 25% kenaf. It was found that the hybrid composites could withstand up to 40 J of impact energy. The damaged area was evaluated by dye penetration testing. Less damaged samples showed higher compressive strength analyzed by compression after impact testing.

##### Jute/Glass

Mohan et al. [72] studied the compressive strength of jute/glass hybrid fiber composites. The aim was to improve the strength and modulus of the jute fiber reinforced plastics. Unidirectional hybrid laminates of glass, epoxy resin, and jute fibers were prepared by filament winding on a flat plate mandrel. The compressive strength of the hybrid composites was lower than the expected value. The micrograph analysis of the failed specimens indicated that the fiber–matrix interface in the hybrid composites was weak and it was confirmed by the low strength values of the hybrid composites. They also suggested that further investigations are needed to understand the performance of the composites.

Clark et al. [73] studied hybrid laminates of randomly oriented jute fiber mats and woven glass fabrics in a polyester resin matrix. The composites were prepared by hand layup method. A variety of laminate constructions were prepared and tested for their mechanical performance and environmental stability. They used a modified rule of mixtures to predict the tensile properties and it was observed that the jute plies control the failure of hybrid laminates at about 0.8% strain. The fractures toughness analysis showed that the maximum toughness is achieved when jute plies are sandwiched between glass fabrics. The impact analysis showed that all the hybrid laminates were tough, although the fabric plies used as the core maximize the work of fracture at about 45 kJ/m^2^. The hybrid laminates with jute facings were unable to withstand the moist environment. Composites were also tested by optical and scanning electron microscopy to explain their mechanical and environmental resistance.

Varma et al. [74] manufactured glass and jute fabrics as reinforcement in unsaturated polyester resin. The fabrics were modified by treatment with ɣ-aminopropyl trimethoxy silane (silane), isopropyl triisostearoyl titanate (titanate), and toluene diisocyanate. The composites were tested for their tensile and flexural properties as well as for interlaminar shear stress. The laminates with jute fabric treated with titanate showed improved mechanical properties and better property retention in a humid environment.

Abdullah et al. [75] presented their work based on hybrid composites of jute and glass mats as reinforcement in unsaturated polyester resin. They reported improved mechanical properties by the addition of glass fibers in the jute fiber reinforced composites. The composite with a jute to glass ratio of 1:3 was best in the mechanical performance. They showed an increase of 125% in tensile strength, 49% in modulus, 162% in flexural strength, and 235% in flexural modulus over the jute composites. The glass and carbon fibers were treated under UV radiation of various intensities, as well. The properties of treated fibers hybrid composites were higher and impact strength was nearly equal to that of only glass fiber reinforced composites. The composites were suggested for applications in housing and automotive interior based on the low-cost UV treatment for surface modification and a good balance of mechanical properties by hybridization.

Ahmad et al. [76] studied the hybrid composites of jute and glass fibers as woven mats in the polyester matrix. The composites were prepared by hand layup method at room temperature. The composites were studied for their impact, tensile, flexural, interlaminar shear strength, and water resistance properties. It was found that the addition of glass fibers to jute fiber composites improved all the measured properties.

Braga et al. [77] described the investigations on hybrid composites of jute and glass fibers in an epoxy matrix. The analysis of the composites showed a significant improvement of the tensile, flexural, and impact strength with a decrement in the moisture absorption for the hybrid composite.

Table 4 highlights the research contributions about the hybrid bast/glass composites, their manufacturing methods, and properties analyzed.

#### 1.3.3. Hybrid Composites of Bast/Basalt Fibers

Hybrid composites of bast/basalt fibers provide an excellent opportunity to improve the mechanical property profile of bast fiber reinforced composites without reducing the amount of their natural content. Adding natural inorganic basalt fibers to the bast fiber reinforced polymer composites means improving the performance of the composite naturally. This section provides an overview of the research contributions about bast/basalt hybrid composites.

##### Flax/Basalt

Boria [78] investigated the experimental study to model the falling weight impact properties of thermosetting composites. Despite the complexity in the model, it was possible to predict the contact forces and final deformation of the composites. The composites were manufactured using a partially bio-based vinyl ester resin with flax and basalt reinforcement by hand layup and resin infusion. The curing was done in an autoclave by applying heat and pressure. The composites were tested for their tensile and flexural properties. The falling weight impact tests were done at various energies up to 40 J to get the data of evolution and the characteristics of damage produced. The tensile performance showed a significant improvement when the flax fiber layers are used as the core between basalt fibers. The advantage of this combination was that it reduced the stiffness and brittleness of the basalt fibers. This observation was also supported by the damage analysis after impact testing. The damage in the hybrid composite appeared to be much less brittle compared to the basalt laminate.

Bakare et al. [79] synthesized a low viscosity bio-based resin from lactic acid, allyl alcohol and pentaerythritol. The composites were produced by resin impregnation and compression molding. The tensile, flexural, Charpy impact, dynamic mechanical, and thermogravimetric properties of the composites were studied. It was found that the hybrid basalt/flax composites had better mechanical properties than the individual flax composites.

Živković et al. [35] studied the hybrid composites of basalt and flax in a vinyl ester matrix. The composites were prepared by hand layup process. The mechanism of energy absorption was studied by SEM analysis of the damaged surface and cross-section. It was found that the hybrid composites had significantly improved impact properties compared to the single flax fiber composite.

Almansour et al. [80] studied the influence of the water absorption on the interlaminar fracture toughness of flax and flax/basalt hybrid composites of vinyl ester. The composites were prepared by vacuum-assisted resin infusion technique. The morphology of delamination and the fracture shear failure of composite laminates were evaluated by scanning electron microscopy and X-ray micro-computed tomography. The experimental results confirmed that the durability and water repellence behavior of flax fiber reinforced composite was enhanced significantly by hybridization with basalt fibers.

Ricciardi [81] investigated the effect of the stacking sequence at the impact damage mechanism, and flexural and interlaminar strength of flax/basalt hybrid epoxy composites. Three different symmetrical configurations of both fibers with almost the same area weights were stacked. The analysis showed that there was no influence of the fiber sequence in the flexural modulus. However, the flexural and interlaminar shear strength exhibited some differences depending upon the resin content. The impact tests were performed at different energies and the damage propagation was characterized. It was concluded that the stacking sequence is relevant in the composite design to meet the specific requirements.

Sarasini et al. [82] manufactured laminates with a hybrid intraply woven fabric based on flax and basalt fibers. The aim was to improve the low-velocity impact response of natural fiber composite. They used both thermoset (epoxy) and thermoplastic matrices (PP with and without malleated coupling agent) for the production of the laminates. It was observed that the energy absorbed and the damage mechanism is significantly affected by the matrix used. The absorbed energy at perforation for PP composites was 90% and 50% higher than that of epoxy composites and compatibilized PP composites, respectively. The influence of low transverse strength of flax fibers on impact response was counteracted by the hybrid fiber structure, irrespective of the matrix type. Thermoplastic laminates showed better quasi-static properties, energy absorption, peak force, and perforation energy compared to the epoxy-based composites. This is because the thermoplastic matrix plasticization delayed the onset of damage during impact.

##### Hemp/Basalt

Ozturk [83] studied the mechanical properties such as tensile, flexural and impact strength of phenol formaldehyde composites with hemp, basalt, and hybrid hemp/basalt as a function of the fiber loading. By the addition of basalt fibers up to 32% by volume, the tensile strength increased. The flexural strength decreased linearly with the increasing fiber content. Impact strength increased up to 48% of the basalt content. The tensile strength of the hemp composites decreased by the addition of basalt fibers. The hemp and basalt composites were fabricated with varying fiber content from 32–63 vol %. The hybrid composites had varying ratios of hemp/basalt fibers with a total fiber loading of 40 vol %. In the case of hemp composites, the tensile strength and elongation at break increased up to 40 vol %, flexural properties increased up to 48% of fiber loading and the impact properties showed a regular trend with an increasing fiber loading. The properties such as tensile, flexural, and Charpy impact strength were better for hemp composites compared to the basalt composites. The mechanical performance increased in the hybrid composites up to 32 vol % of the basalt fibers. The maximum flexural strength was shown by the composite with 0.52:0.48 hemp/basalt ratio (122 MPa) and the maximum impact strength (~27 kJ/m^2^) was obtained for 0.68:0.32 hemp/basalt fiber ratio.

Czigány [84] presented the mechanical analysis and acoustic emission tests of PP composites with basalt (BF), hemp (HF), glass (GF), and carbon fibers (CF) as single fiber and hybrid composites. There were eleven compositions abbreviated as L1–L11 as shown in Table 5.

The aim of this paper was not really the improvement of mechanical performance of bast fibers in hybridization with basalt fibers, rather it was a comparative study of the hybrid composites of basalt fibers with hemp, glass, and carbon fibers and it was concluded that hybrid composites with glass and carbon had superior properties. The composites were prepared by carding, needle-punching, and press molding. For improved fiber–matrix adhesion, the polypropylene fibers were treated with a product of the addition reaction of a mixture of sunflower oil and maleic anhydride. The hybrid effect was examined as a function of fiber content and combination. The strength properties of the hybrid composites with surface treatment were better than the composites without treated fibers. The hybrid composites of basalt with hemp, carbon, and glass were compared for their mechanical properties. The study revealed that the hybrid basalt/hemp composites manifested only a slight increase in the mechanical properties, while it was significant in the case of basalt/carbon and basalt/glass fiber hybrid composites. This led to the conclusion that inexpensive basalt fibers can be applied in carbon and glass hybrid composites in an efficient way for cost saving. Compared to the pure PP matrix, the mechanical properties improved in each case, both for single and hybrid composites. A correlation was found between the characteristics of the ultrasound signals that occurred during loading the hybrid composites and the mechanical properties of the examined composites that made it possible to monitor the state of the composite material structure during production and to estimate its remaining stress.

Dhakal et al. [85] developed hemp fiber composites in unsaturated polyester resin and studied the influence of hybridization at post-impact behavior and damage tolerance capability of hemp fiber composites. The laminates were impacted at 3, 6, and 9 J energy in both quasi-static and cyclic flexural tests with a step loading procedure. The acoustic emission tests confirmed that there are several limitations in the use of natural fiber reinforced composites even if the impact energy is not so close to penetration. The damage tolerance was significantly improved for the hybrid composites of basalt/hemp.

Sarasini et al. [86] prepared hemp, basalt and hemp/basalt hybrid composites from high-density polyethylene by injection molding. The mechanical properties of the hybrid composites increased significantly compared to the composite with only hemp fiber, making it possible to use these materials in semi-structural applications. Additionally, they demonstrated a procedure for sizing removal mimicking the conditions experienced in an end-of-life composite thermal recycling process and discussed it in terms of residual mechanical properties of basalt/HDPE composites.

Sergi et al. [87] studied the hybrid hemp/basalt composites of high-density polyethylene (HDPE) with a special focus on the effects of hygrothermal UV radiation aging. The HDPE was modified with maleic anhydride high density polyethylene copolymer. The water uptake in the hybrid composites decreased considerably and the retention of mechanical properties after accelerated aging increased.

Kumar et al. [88] investigated the influence of temperature and hybridization on the impact damage evolution and post-impact residual strength of hemp/epoxy, basalt/epoxy and their hybrid composites. The composites were analyzed for their mechanical and acoustic emission properties. At first, the samples were studied by drop weight impact testing at 30 °C and 65 °C. After that, they were studied for their flexural properties for the assessment of residual strength, whereas the acoustic emission signals were recorded during the test. The analysis of results showed that the hybridized laminates have better resistance to impact at 65 °C. At ambient temperature (30 °C) basalt/epoxy laminates showed high impact damage.

Petrucci et al. [89] investigated hybrid composites laminates of basalt, glass, flax, and hemp prepared by vacuum infusion. All laminates had a fiber volume fraction of 21–23% in an epoxy resin. The mechanical properties were characterized by tensile, three-point bending and interlaminar shear strength. The fracture surfaces were characterized by scanning electron microscopy. The analysis showed that the mechanical properties of the hybrid laminates were better than the hemp and flax fiber reinforced laminates and were lower than the basalt fiber laminates. The best performance was shown by the hybrid glass and flax with basalt laminates. In a further contribution, Petrucci et al. [90] studied the impact and flexural properties of hybrid composites of basalt, flax, hemp, and glass in epoxy resin. The reinforcement was about 21–23% in all composites. The impact performance of glass/hemp/basalt composites was worse than the flax/hemp/basalt laminates. The best impact penetration energy was shown by flax, hemp, and basalt laminate. The composites were also studied by scanning electron microscopy and acoustic emission.

##### Kenaf/Basalt

Mokhtar [91] studied the tensile, flexural, and impact strength properties of kenaf, basalt, and hybrid kenaf/basalt composites of a blend of ultra-high molecular weight polyethylene/high-density polyethylene matrix. The composites were prepared by melt compounding in extrusion. The analysis showed the high elastic modulus for basalt fiber composites (1 GPa) compared to the kenaf and hybrid kenaf/basalt hybrid composites (0.9 MPa). The tensile performance of the composites was much better in comparison with the other properties. The composites without kenaf showed low flexural strength (27 MPa) and Charpy strength properties.

Umashankaran et al. [92] manufactured hybrid basalt/kenaf epoxy composites using a hand layup technique. The composites were made and compared at four sequence combinations, hybrid composites with three sack-layers and individual three-sack layered basalt and kenaf composites. The composites were subjected to tensile and flexural analysis and the fracture surface was analyzed by SEM to identify the failure mode. The mechanical analysis showed the maximum tensile strength of 111Mpa and a maximum flexural strength of 283.9 MPa for the hybrid basalt/kenaf composites.

Atiqah et al. [93] studied the damping properties of kenaf/basalt epoxy hybrid composites by dynamic mechanical analysis. Composites were prepared by the vacuum infusion method. The study aimed to investigate the viscoelastic behavior of hybrid kenaf/basalt epoxy composites. The highest damping properties were achieved with basalt fiber composites. The damping behavior of the hybrid composite was intermediate between pure basalt and pure kenaf composites.

**Figure 4:**
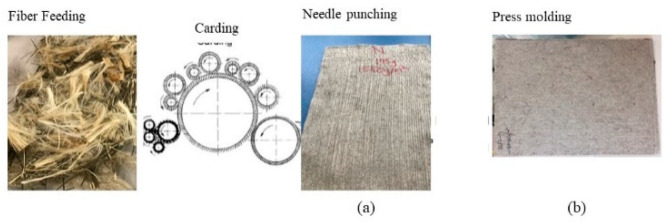
Schematic representation of composite manufacture by carding, needle punching, and subsequent compression molding (**a**) needled fiber mat, (**b**) laminate after hot pressing (modified illustration [84,94,95]).

Most of the work reported about the manufacture of hybrid bast/basalt composites involves vacuum assisted resin transfer and hand layup methods. Very few contributions have shown the hybrid composites manufactured by carding, needling, and compression molding. Saleem et al. [94,95] studied the hybrid composites of bast and basalt fibers in polyester-based thermoset and thermoplastic polypropylene composites. They used flax, kenaf, hemp, and a mixture of flax and kenaf as the bast fibers. The composites were tested for their tensile, flexural, Charpy impact, and thermal properties. The production of thermoplastic composites involved initially the production of fiber mats of matrix, basalt, and bast fibers by carding and needling (Figure 4). The needled fiber mats were compression molded and the laminates were characterized for their mechanical, morphological, and thermal properties.

The thermoplastic composites were prepared by carding the bast and basalt fibers followed by the needling of the fiber mass. The needled fiber mass was further impregnated by the resin immersion technique using a foulard. These impregnated mats after drying were compression molded to the laminates. The laminates were characterized further for their mechanical and morphological properties. The properties were significantly improved in the case of hybrid bast/basalt composites. It was found that the basalt fiber sizing or polymer modification were important parameters to optimize the mechanical performance of the composites by improving their fiber–matrix interaction.

##### Jute/Basalt

Amuthakhanan et al. [96] studied various stacking sequences of basalt and jute fibers in hybrid composites prepared by compression molding. The composites were analyzed for their tensile, flexural, and impact behavior properties. It was observed that the tensile and flexural properties were better for the composites having alternating layers of basalt and jute fibers. SEM was used to study the fracture morphology.

Parasath et al. [97] manufactured composites of basalt and jute fabric in a polyester matrix by compression molding. The composites were prepared in various stacking sequences. They were analyzed for their tensile, flexural, and Charpy impact properties. It was concluded that the hybrid basalt composites have higher tensile and flexural properties than the jute composites. Whereas the jute fiber composites are better impact properties.

Fiore et al. [98] studied the aging resistance of jute/basalt interply hybrid laminates. The aim was to investigate the possibility to enhance the durability of natural fiber reinforced composites for marine applications. The composites were manufactured by vacuum-assisted resin infusion. The influence of the aging time on the mechanical properties was studied by flexural and low-velocity impact analysis. It was found that the substitution of the external jute layer with basalt layer (sandwich-like arrangement) gave the best results to enhance the durability of the composites exposed to salt-fog conditions.

The data is scarce about the long-term durability of natural fiber reinforced composites. Considering this Ma et al. [99] studied the effect of water, alkali and temperature on the weight gain, tensile properties, and surface morphology of hybrid jute/basalt epoxy composites. Both treated (with alkali and silane) and untreated jute fibers were used. It was concluded that the weight gain was higher for jute composites. Both alkali and silane treatment of the jute fibers reduced the water absorption and enhanced the tensile strength of the composites. Prolonged exposure to temperature, water, or alkali solution reduced the strength of all the composites. However, the basalt fiber composites were better in performance.

Gangapa et al. [100] manufactured hybrid jute/basalt composites to combine the high tensile and modulus properties of basalt with antistatic properties of jute in polyester resin. The composites were manufactured by hand layup and compression molding technique and tested for their tensile and flexural properties in warp and weft direction. They compared the results of the 2- and 3-mm thick hybrid composites with each other. The tensile properties of 3 mm thick sample were less than the 2 mm thick samples. Whereas, 3 mm thick sample had higher compression strength. This increase and decrease of the properties were attributed to the variation in fiber reinforcement. The dynamic mechanical study showed that the damping ratio of 2 mm sample was 5.78 % higher than the 3 mm thick sample. They concluded that it could be due to the difference in stiffness of the laminate because of variations in thickness and volume fraction.

Table 6 presents an overview of the manufacturing methods and the property evaluation of hybrid bast/basalt composites.

### 1.4. Applications of Hybrid Composites

The most widely used composites in automotive industry are glass fibers reinforced composites. Hybridization provides an opportunity to substitute partially, if not fully the glass fiber content of the composites with natural fibers. Hybrid kenaf/glass fiber composites have shown properties comparable to the glass fiber composite as brake lever in automotive [101]. Furthermore, natural/glass fiber hybrid composites have manifested reduced moisture absorption compared to the natural fiber composites increasing their chances in structural applications [68,102,103]. Flax/glass hybrid composite in epoxy was studied for marine applications and showed enhanced aging durability in marine environmental conditions [63].

Basalt fibers are considered a natural alternative of the glass fibers. Hybridizing basalt fibers with bast provides a completely natural alternative to the hybrid bast/glass fiber composites. Basalt fibers other than providing enhanced mechanical strength and environmental benefits, improve the high-temperature resistance of the composites. The companies Expancore Composite Technologies in Sweden and Fibtex GmbH in Germany have developed a noncombustible basalt fiber mat which was presented at Composites Europe 2019 exhibition [104]. In addition to the mechanical properties of the hybrid composites, safety is an important aspect to be considered while using them in a vehicle. An important parameter for safety is the inflammability of the materials. Basalt fibers, along with enhancing the mechanical property profile of the composites, have the potential to improve their inflammability characteristics [105].

In the automotive sector, their assignment in structural applications is gaining more and more attraction. International Automotive Components in cooperation with BASF Corporation, Ludwigshafen, Germany launched a natural fiber roof frame for 2017 Mercedes Benz E-Class. It is based on 70% natural fibers and provides up to 50 % weight reduction compared to the conventional metal-reinforced sunroofs [106]. The efforts [4,67,100,102,103] to partially replace conventional fiber reinforced composites with better performing and environmentally-friendly materials are evident from the literature review [107,108,109]. The demand of such composites is almost constant in the automotive industry because of their suitable properties for this sector such as low density, CO_2_ footprint, and good crash behavior [110]. Their use in other applications is also attractive and efforts are ongoing to apply them in, for example in the biomedical area [40] or even construction sector, as well.

## 2. Conclusions

The use of plant (bast) fiber-reinforced polymer composites, predominantly in semi-structural automotive applications, is well established. The advantages they offer in this sector are lower weight and cost reduction. They have better recycling capabilities, sustainability, and environmental friendliness compared to synthetic fiber composites. However, in structural applications, their use is limited. This is because of their poor mechanical performance that cannot compete with the dominant reinforcement fibers on the market such as carbon or glass. Hybridization of natural fibers with glass, carbon, or basalt is a good opportunity to manufacture bast fiber composites with enhanced mechanical properties. It is a very good possibility to overcome the limitations of bast fiber composites in structural applications. However, the issues with natural fibers such as naturally occurring irregularities or moisture absorption and their incompatibility with certain matrices have to be handled as well, so that the benefits of hybridization could be achieved.

This contribution provides an overview of the research work about the hybrid composites of bio-based bast fibers with carbon, glass, and basalt fibers in polymer composites. Their property evaluation, individual reinforcements or matrix modification and processing methods have been reviewed. The main findings of the review are:

1. Hybridization improves the properties of hybrid composites compared to the conventional single bast fiber composites and is an effective method that can be employed to tailor properties of bast fiber-reinforced composites for structural applications.
2. Hybrid composites of bast fibers with high strength fibers show a simultaneous increase in mechanical property profile and reduction of water absorption.
3. Fiber–matrix interaction is very important in defining the mechanical properties. In most cases, mechanical properties increase by fiber or polymer modification.
4. The fiber layers and their stacking sequence have a significant influence on the mechanical performance.
5. Hybridization with basalt fibers instead of carbon or glass provides a more sustainable alternative for the improvement of mechanical properties because of the environmental friendliness and sustainable properties of basalt fibers.
6. Most of the reviewed literature is about the physical and mechanical properties, use of coupling agents or fiber modification, and hand layup or vacuum-assisted resin transfer manufacturing methods of hybrid composites. Very few contributions have studied the thermal, electrical, and damping properties of these composites and manufacturing methods for rapid production in commercial applications like carding, needling, injection molding, extrusion, or resin impregnation by immersion.

It is recommended that future research in this field should not only be driven by the automotive applications of hybrid bast composites, but also their applications in other important fields such as construction and biomedical need exploration. Modern analysis methods, such as X-ray photoelectron spectroscopy or atomic force microscopy and analytical models, are required for the optimization of their widespread applications.

## Figures and Tables

**Table 1:** Mechanical properties and sustainability characteristics of carbon, glass, basalt, and bast fibers [3,15,16,17]. The cost is based on public information [18].

Fiber	Density	Diameter	Tensile Strength	Tensile Modulus	Cost [18]	Elongation at Break	Renewability	Health Risks
	(g/cm^3^)	(µm)	(MPa)	(MPa)	(€/kg)	(%)		
Carbon	1.80	5–10	2000–5000	200–600	26–34	1.5–2	No	Yes
Glass	2.50	5–25	1700–3500	65–72	0.42–2.56	2.5	No	Yes
Basalt	1.40	10–20	2800–3100	80–90	0.34–3.42	3.1	Yes	No
Flax	1.2–1.5	12–20	400–600	12–25	1.3–1.4	1.2–1.6	Yes	No
Hemp	1.3–1.5	25–500	300–700	20–70	5–10	1.6	Yes	No
Kenaf	1.1–1.2	30–40	150–250	10–20	1–3	2.7–6.9	Yes	No
Jute	1.3–1.5	17–20	350–780	20–30	1.2–1.6	1.8	Yes	No

**Table 2:** Chemical composition of basalt and glass fibers [29].

Element	Oxide	Basalt Fibers		Glass Fibers	
		Element	Oxide	Element	Oxide
		(m%)	(m%)	(m%)	(m%)
Al	Al_2_O_3_	9.17	17.35	6,3	11.86
Si	SiO_2_	19.76	42.43	27.24	58.25
Ca	CaO	6.35	8.88	15.05	21.09
Fe	Fe_2_O_3_	8.17	11.68	0.21	0.30
K	K_2_O	1.94	2.33	0.36	0.43
Mg	MgO	5.70	9.45	0.32	0.54
Na	Na_2_O	2.81	3.67	0.22	0.30
Ti	TiO_2_	1.53	2.55	0.25	0.41

**Table 3:** Research contributions about hybrid bast/carbon fibers composites.

	Bast Fiber	Matrix	Fiber or Matrix Modification	Composite Manufacturing Technique	Evaluation
[7]	Flax	Epoxy	-	Vacuum bagging	Three-point bending, tensile
[40]	Flax	Epoxy	-	Resin impregnation, compression molding	Three-point bending, tensile, Rockwell hardness
[41]	Flax	Epoxy	-	Resin impregnation, compression molding	Water absorption technique, flexural, tensile, and thermogravimetry
[42]	Flax	Epoxy	-	Thermopressing	Damping
[43]	Flax	Epoxy	-	Compression molding and vacuum bagging	Damping, flexural, tensile
[44]	Flax + Basalt	Epoxy	-	Hand layup and vacuum bagging	Tensile, flexural, damping, interlaminar shear strength, impact
[39]	Flax	Epoxy	-	Vacuum bagging process	Low speed impact testing, flexural properties
[47]	Hemp	unsaturated Polyester	-	Resin impregnation	Impact testing (low speed)
[48]	Hemp	Polyester	Alkali treated hemp fibers	Hand layup	Water absorption, flexural, tensile and impact
[49]	Hemp	Epoxy	-	Hand layup and vacuum compression molding	Tensile, flexural, falling weight impact testing, interlaminar shear stress
[57]	Kenaf	Thermoplastic natural rubber	Sulfuric acid treated carbon fibers, Maleic anhydride grafted polypropylene (MAPP)	Compounding in an internal mixer	Flexural, tensile, impact analysis, dynamic mechanical analysis
[50]	Kenaf	Epoxy	Sodium hydroxide treated Kenaf fibers, Gamma radiations treated carbon fibers	Resin transfer and compression molding	Impact analysis
[51]	Kenaf	Epoxy	-	Vacuum bagging	Tensile and compression analysis
[52]	Kenaf	Epoxy	-	Vacuum infusion	Tensile, flexural and impact analysis
[53]	Jute	Epoxy	-	Hand layup	Moisture content, impact analysis
[54]	Jute	Epoxy	-	Resin transfer molding	Tensile, dynamic mechanical, optical and surface analysis
[55]	Jute	Polyester	-	Vacuum bagging process	Dynamic mechanical analysis
[56]	Jute	Epoxy	-	Hand layup	Tensile, flexural, and impact analysis

**Table 4:** Research contributions about hybrid bast/glass fiber composites.

Reference	Bast Fiber	Matrix	Fiber or Matrix Modification	Composite Manufacturing Technique	Evaluation
[59]	Flax	PP	**-**	Co-needling of discontinuous fibers followed by hot pressing	Dynamic mechanical analysis, thermal analysis, Charpy impact and falling weight impact, SEM
[60]	Flax	Acrylated epoxidized soybean oil	Untreated Flax Fibers Treated flax fibers with sodium hydroxide flax fibers	Resin transfer	Compression, flexural, drop weight impact, water absorption,
[23]	Flax	Phenolic resin	-	Compression molding	Tensile analysis, interlaminar shear stress
[61]	Flax	PP	Maleic anhydride grafted PP (MAPP)	Compounding in twin screw extruder	Water absorption, thermal aging, UV ageing, tensile, impact, hardness analysis, SEM
[62]	Flax	Epoxy	-	Compression molding	Water absorption, tensile testing, acoustic emission, damage mechanism assessment
[63]	Flax	Epoxy	-	Vacuum assisted resin infusion	Salt-fog aging test, water absorption, wettability, flexural, dynamic mechanical analysis, SEM
[64]	Flax	Vinyl ester	Flax fibers treated with sodium hydroxide	Vacuum assisted resin transfer	Tensile, flexural, impact, water absorption, thermogravimetric analysis
[65]	Flax	Vinyl ester	-	Hand layup, Vacuum assisted resin infusion	Moisture absorption, low velocity falling weight impact test, impact damage characterization by SEM and X-ray micro CT
[66]	Hemp	PP	-	Melt compounding	Fiber length measurement, tensile, flexural analysis, water absorption, heat deflection temperature, thermogravimetric analysis, SEM
[67]	Hemp	Unsaturated polyester	-	Hand layup, compression molding	Tensile, low velocity impact, fatigue
[68]	Kenaf	Epoxy	-	Modified sheet molding compound	Tensile, flexural, impact, and SEM analysis
[69]	Kenaf	Unsaturated polyester	Sodium hydroxide treated kenaf fibers	Modified sheet molding compound process	Flexural and impact analysis
[70]	Kenaf	Unsaturated polyester	Sodium hydroxide treated kenaf fibers	Sheet molding process	Flexural and fracture analysis
[71]	Kenaf	Epoxy	-	Hand layup	Low velocity impact, dye penetrant, compression after impact analysis
[72]	Jute	Epoxy	-	Filament winding	Compressive properties, micrograph analysis of the failure
[73]	Jute	polyester	-	Hand layup	Tensile, fracture toughness, Impact analysis
[74]	Jute	Unsaturated polyester	Titanate, silane treated jute fibers	Hand layup, Ccompression molding	Tensile, flexural, effect of humidity
[75]	Jute	Unsaturated polyester	-	Hand layup	Tensile, Chapy impact, SEM analysis
[76]	Jute	Polyester	-	Hand layup	Tension, tensile, flexural, and interlaminar shear analysis
[77]	Jute	Epoxy	-	Hand layup	Flexural, tensile, impact, density and water absorption analysis, thermogravimetric analysis

**Table 5:** Compositions of the prepared composites L1–L11 showing the fiber content in wt % (modified table [84]).

	L1	L2	L3	L4	L5	L6	L7	L8	L9	L10	L11
PP	100	83	75	74	70	79	79	77	76	76	73
BF	-	17	-	-	-	12	12	12	6	6	6
HF	-	-	25	-	-	9	-	-	18	-	-
GF	-	-	-	26	-	-	9	-	-	18	-
CF	-	-	-	-	30	-	-	11	-	-	21

**Table 6:** Research contributions about hybrid bast/basalt composites.

Reference	Bast Fiber	Matrix	Fiber or Matrix Modification	Composite Manufacturing Technique	Evaluation
[78]	Flax	Vinylester	-	Hand layup, resin infusion	Tensile, flexural, impact falling weight, and SEM analysis
[79]	Flax	Low viscosity biobased resin from lactic acid = PMLA Resin	-	Hand layup	Tensile, flexural, Charpy impact, and water analysis
[35]	Flax	Vinylester	-	Hand layup	SEM, low velocity impact analysis
[80]	Flax	Vinylester	-	Vacuum infusion	Water absorption, interlaminar fracture toughness, SEM, X-ray computed micro-tomography, fracture energy,
[81]	Flax	Epoxy	-	Vacuum infusion	Interlaminar shear stress, flexural, tensile, impact, fractography
[82]	Flax	Epoxy, polypropylene	Maleic anhydride grafted polypropylene (MAPP)	Vacuum infusion, compression molding	Tensile, flexural, drop weight impact, morphology, and damage investigations
[83]	Hemp	Phenol formaldehyde	-	Compression molding	Tensile, flexural, and Charpy impact analysis and SEM
[84]	Hemp	Polypropylene	Sunflower oil and maleic anhydride treated fibers	Carding, needling and compression molding	Tensile, flexural, acoustic emission analysis
[85]	Hemp	Unsaturated polyester	-	Hand lay-up and compression molding	Low velocity impact testing, flexural analysis, acoustic emission, SEM
[86]	Hemp	High density polyethylene	-	Melt compounding and injection molding	X-ray photoelectron spectroscopy, SEM, tensile testing, differential scanning calorimetry, fiber aspect ratio, Vicat softening temperature, influence of basalt fiber sizing at mechanical properties
[87]	Hemp	High density polyethylene	HDPE modified by maleic anhydride high density polyethylene copolymer	Melt compounding and injection molding	Water absorption, tensile, SEM, accelerated ageing
[88]	Hemp	Epoxy	-	Hand layup and compression molding	Impact and flexural analysis with acoustic emission monitoring
[89]	Flax/hemp	Epoxy	-	Vacuum infusion	Tensile, flexural, interlaminar shear stress and SEM
[90]	Flax/hemp	Epoxy	-	Vacuum infusion	Impact, acoustic emission analysis
[91]	Kenaf	blend of thermoplastic polyethylene (UHMWPE/HDPE)	-	Melt compounding, compression molding	Flexural analysis, Charpy impact, and tensile
[92]	Kenaf	Epoxy	-	Hand layup, compression molding	Tensile, flexural, fracture analysis by SEM
[93]	Kenaf	Epoxy	-	Vacuum infusion	Dynamic mechanical analysis
[96]	Jute	Polyester	-	Compression molding	Tensile, flexural, impact
[97]	Jute	Polyester	-	Compression molding	Tensile, flexural, impact
[98]	Jute	Epoxy	-	Compression molding	Aging resistance by flexural and low velocity impact response
[99]	Jute	Epoxy	Silane and alkali treated jute fibers	Vacuum assisted resin infusion	Water absorption, tensile
[100]	Jute	Polyester	-	Hand layup and compression molding	Tensile, compressive and dynamic properties
